# Specular highlights improve color constancy when other cues are weakened

**DOI:** 10.1167/jov.20.12.4

**Published:** 2020-11-10

**Authors:** Rebecca Wedge-Roberts, Stacey Aston, Ulrik Beierholm, Robert Kentridge, Anya Hurlbert, Marko Nardini, Maria Olkkonen

**Affiliations:** 1 Department of Psychology, Durham University, Durham, UK; 2 Neuroscience, Institute of Biosciences, Newcastle University, Newcastle, UK; 3 Department of Psychology and Logopedics, Faculty of Medicine, University of Helsinki, Helsinki, Finland; 4 Azrieli Programme in Brain, Mind & Consciousnesses, Canadian Institute for Advanced Research, Toronto, Canada

**Keywords:** color constancy, specular highlights, cue combination, daylight prior

## Abstract

Previous studies suggest that to achieve color constancy, the human visual system makes use of multiple cues, including a priori assumptions about the illumination (“daylight priors”). Specular highlights have been proposed to aid constancy, but the evidence for their usefulness is mixed. Here, we used a novel cue-combination approach to test whether the presence of specular highlights or the validity of a daylight prior improves illumination chromaticity estimates, inferred from achromatic settings, to determine whether and under which conditions either cue contributes to color constancy. Observers made achromatic settings within three-dimensional rendered scenes containing matte or glossy shapes, illuminated by either daylight or nondaylight illuminations. We assessed both the variability of these settings and their accuracy, in terms of the standard color constancy index (CCI). When a spectrally uniform background was present, neither CCIs nor variability improved with specular highlights or daylight illuminants (Experiment 1). When a Mondrian background was introduced, CCIs decreased overall but were higher for scenes containing glossy, as opposed to matte, shapes (Experiments 2 and 3). There was no overall reduction in variability of settings and no benefit for scenes illuminated by daylights. Taken together, these results suggest that the human visual system indeed uses specular highlights to improve color constancy but only when other cues, such as from the local surround, are weakened.

## Introduction

### General overview

Observers generally have little difficulty judging objects as having a relatively stable color under changes in illumination—an ability termed color constancy. However, it is still unknown to what extent they are able to make use of specular highlights to assist in this. In this series of experiments, we tested whether observers’ color constancy improves when specular highlights are present. We also investigated whether judgments are less variable and more accurate along the daylight locus, consistent with the use of daylight priors, and whether these would interact with specular highlights.

### Color constancy and cue use

**Figure 1.**
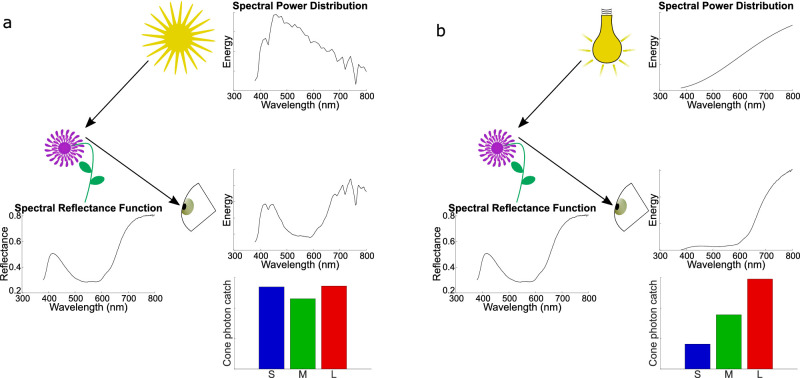
(a) Spectra of light reaching observer from a flower under D65. (b) Spectra of light reaching observer from the same flower under Illuminant A. Relative cone activations for each scenario are shown at the bottom.

Perceiving objects to have a stable surface color under changing illuminations is a remarkable computational challenge that our brains solve every day. This problem is demonstrated in Figure 1. In Figure 1a, a purple flower is seen outside under daylight (D65). The light reaching an observer's eye is the product of the spectral power distribution of the illumination and the spectral reflectance of the flower. Moving the flower inside under a tungsten light bulb (Illuminant A; see Figure 1b) changes the spectrum of light reaching the observer's eye. While this changes the relative photoreceptor activation in the retina, our observer would probably not judge the flower to have changed color appreciably. The extent to which the color appearance of the flower changes depends on multiple factors, including the extremeness of the illumination change, the duration of exposure and adaptation to each illumination, the surface properties of the flower, and its context. Yet, in everyday life, people would tend to attribute a constant color to the flower despite changes in the light it reflects caused by changes in illumination.

As the signal reaching the eye confounds illumination with reflectance information, our brains must compensate for any change in illumination in order to create stable perceptions corresponding to the object's invariant surface reflectance properties. This is computationally difficult as an infinite number of combinations of reflectances and illuminations can yield the same photoreceptor activation, and surfaces that are metamers (appear identical) under one illumination may not be metamers under a different illumination (Logvinenko, Funt, Mirzaei, & Tokunaga, 2015). However, given the constraints of natural illuminations and surfaces, the problem becomes more tractable (see Hurlbert, 1998, for a review of computational models).

Many computational color constancy algorithms have been proposed that all incorporate some form of illumination estimation from regularities in the input image (for reviews, see Smithson, 2005; Lee & Plataniotis, 2014). Although most of the algorithms have not been explicitly proposed or tested experimentally as models of human visual perception, a small number of studies suggest that certain proposed cues do influence perceived illumination chromaticity, such as local surround (Valberg & Lange-Malecki, 1990), the global mean chromaticity (“gray world”) (Land, 1983, 1986), or the chromaticity of the brightest surface (e.g., “brightest is white”; Hurlbert, 1998). Kraft and Brainard (1999) demonstrated that these three cues can all be used in combination and that none of them is alone sufficient to account for color constancy performance.

**Figure 2.**
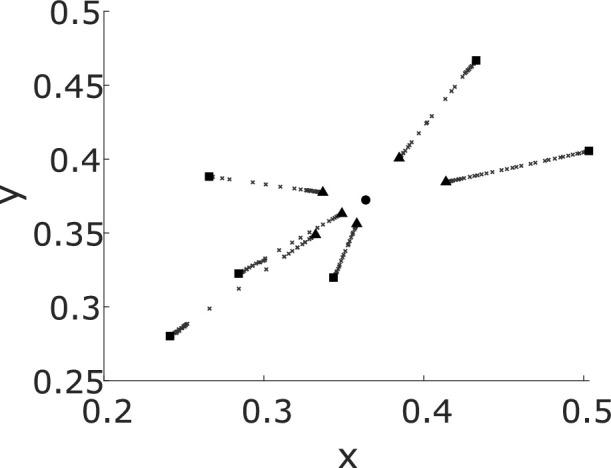
Chromaticity in CIE xy color space, taken from six objects with different reflectances under the same illumination. Gray +s show individual pixel chromaticities and fall on lines between the diffuse reflectance (squares) and specular reflectance (triangles). The black circle shows the illumination chromaticity. As can be seen, the chromaticity of the light reflected from the six objects converges toward the illumination chromaticity.

Specular highlights may also be used to gain information about the illumination chromaticity. Perfectly specular highlights are perfect reflections of the incident illumination and therefore have the same chromaticity as the illumination. However, most objects in the real world are not purely specular. A model proposed by Lee (1986) and D’Zmura and Lennie (1986), coined chromaticity convergence (Hurlbert, 1998), explains how partially specular objects (more precisely, optically inhomogeneous materials) can assist in estimating illumination chromaticity. The light reflected by partially specular objects has two components: a diffuse component, in which light has been scattered throughout the material before being reflected back—typically referred to as diffuse reflectance, or “color”—and a specular component. From a single object, the reflected light is a mixture of these two components, and therefore its chromaticity falls on a line in color space between the diffuse and specular components. When there are two or more surfaces present, these lines would point toward the illumination's chromaticity and, if extended, they would intersect at the chromaticity of the illumination (see Figure 2). It should be noted that the specular highlights do not need to be visible for chromaticity convergence; as long as there are multiple partially specular surfaces with different diffuse components present, the chromaticities will form lines toward the illumination chromaticity.

### Research into the use of specular highlights

In one of the few studies into the use of specular highlights, Yang and Maloney (2001) rendered a set of specular spheres under two illuminations (D65 and A). They then swapped the highlights from Scene A with those in Scene B and vice versa. The observer's task was to adjust the color of a patch in the scene so that it appeared to be gray. These achromatic settings shifted toward the illumination chromaticity when perturbed highlights signaled a daylight illumination (D65), but not when they signaled a nondaylight illumination (A). Furthermore, specular highlights were only used when the test patch was on one of the specular objects in the scene, not when it was on the back wall. This suggests that observers segment scenes into different illumination frameworks, which could be achieved based on the depth plane in which an estimate is being made (see Werner [2006] and Snyder, Doerschner, & Maloney [2005]).

Yang and Shevell (2003) also perturbed specular highlights in an asymmetric matching task under D65 and A and found observers’ color constancy to improve with consistent highlights. Additionally, Yang and Shevell (2002) found better color constancy for scenes containing specular highlights compared to scenes without. Lee and Smithson (2016) found that observers can use specular highlights, even without any other information, for operational color constancy (distinguishing a reflectance change from an illumination change). Xiao, Hurst, MacIntyre, and Brainard (2012) found better color constancy for glossy, compared to matte, shapes but only when other cues were reduced. Measuring lightness constancy, Boyaci, Doerschner, and Maloney (2006) found that some (two out of six) observers could use highlights on spheres in a void to determine an illuminant's direction and brightness. Taken together, these few studies suggest that observers can use specular highlights to improve their color and lightness constancy under certain conditions.

### Cue combination

In non-color-related domains, observers are able to optimally combine multiple cues to improve estimates of ambiguous stimulus properties (Ernst & Banks, 2002; Ernst, 2006), as shown by decreased variable error, compared to estimates made using single cues alone. Optimal integration, according to maximum likelihood estimation and Bayesian models, means that the reliability of an estimate based on multiple cues is the sum of the reliabilities of each individual cue (Van Beers, Sittig, & Gon, 1999; Landy, Maloney, Johnston, & Young, 1995). Thus, having more cues available should increase the reliability, thereby decreasing the variability. So far, none of the research into cue use in color constancy has considered this model of optimal cue integration, instead focusing only on color constancy indices. Here, we ask whether adding a specular highlight cue decreases the variability of achromatic settings in addition to improving color constancy.

### Daylight priors

Bayesian models also predict that combining priors (previous knowledge) with current information should aid in the perception of ambiguous stimuli by increasing accuracy and decreasing variability of estimates (Knill & Richards, 1996). Color constancy may benefit from a prior for daylight illuminations (Brainard, Longére, Delahunt, Freeman, Kraft, & Xiao, 2006). Daylight illuminations are broadband mixtures of sunlight and skylight, and vary in a regular, predictable way. Their chromaticities fall along a curve—the “daylight locus” (Judd et al., 1964; Spitschan, Aguirre, Brainard, & Sweeney, 2017)—in the chromaticity plane, ranging in appearance from orangish to bluish.

Research tentatively supports the notion that observers have a prior for daylights, although the prior seems to be asymmetric such that the improvements are usually found for bluish illuminations. Pearce, Crichton, Mackiewicz, Finlayson, and Hurlbert (2014) found lower sensitivity to changes in illumination along the daylight locus than along the opposite reddish-greenish direction, implying better color constancy for daylight illuminations, especially in the bluish direction (see also Radonjić & Brainard, 2016; Radonjic, Ding, Krieger, Aston, Hurlbert, & Brainard, 2018; Aston, Radonjic, Brainard, & Hurlbert, 2019). Additionally, Weiss, Witzel, and Gegenfurtner (2017), using a different technique (achromatic adjustment), found the highest degree of color constancy under a bluish daylight illumination and achromatic settings made under all other illuminations were skewed toward blue chromaticities. Finally, Delahunt and Brainard (2004) found the highest color constancy for bluish daylight illuminations, but the effect was robust only when the local contrast cue was silenced (also see Brainard et al., 2006). As noted above, Yang and Maloney (2001) found that specular highlights were only used as a cue to the illumination when they signaled a daylight and not when they signaled a tungsten illumination. This, together with the finding by Delahunt and Brainard (2004), suggests that the effect of a daylight prior interacts with the effect of cues, such that the prior is used when cues are weakened or, in the case of Yang and Maloney (2001), conflicting.

### Current study

Taken together, previous research suggests that the visual system may be able to use specular highlights and daylight priors to support color constancy in certain circumstances. However, there are little data on the interaction of a specular highlights cue with a daylight prior. Furthermore, the research into both areas is limited, and much of it lacks the power to conduct formal statistical analyses or to quantify the benefit of either. Therefore, the contexts in which specular highlights may be useful remain unclear. Here, we take a novel cue combination approach to ask whether, in addition to supporting color constancy, specular highlights help decrease variability of illumination estimates. We characterized color constancy with achromatic adjustment and used the equivalent illuminant modeling approach to obtain observers’ illumination estimates for scenes containing matte or glossy shapes, under illuminants either on or off the daylight locus.

### Hypotheses

(1) Specular highlights will be used as a cue to the illumination for color constancy, shown by improved color constancy and decreased variable error for scenes containing specular highlights compared to scenes without.
(2) The effect of specular highlights will be mediated by (a) the type of illumination, such that observers will use highlights more when they signal a daylight (bluish/yellowish, but potentially more so for blues), and (b) position of achromatic matching patch, such that observers will only use the highlights when making settings on a specular object that lies in the same illumination framework.
(3) Observers will show a higher degree of color constancy for scenes illuminated by daylights (particularly blues). This effect will be mediated by the presence of other cues so the daylight prior will be relied upon more for scenes with no specular highlights and an invalid or biased local surround cue.

## Experiment 1

To test our hypotheses, we asked observers to set a patch in a three-dimensional rendered scene to look gray (achromatic adjustment) when the objects were either matte (and therefore had no specular highlights) or when they were glossy (and had a valid specular highlights cue). The scenes were illuminated by either bluish or yellowish illuminants on the daylight locus, or reddish or greenish illuminants off the daylight locus.

### Methods

#### Observers

Fourteen paid volunteers aged between 20 and 36 years (mean age = 24.6; 9 females) took part in this study. All observers were naive to the purpose of the study. All observers had normal color vision, as screened by Ishihara plates (Ishihara, Tokyo, Japan), and all had normal or corrected-to-normal visual acuity.

#### Materials and apparatus

Stimuli were presented on a 10-bit ASUS (Beitou District, Taipei, Taiwan) PA382Q 23-in. monitor controlled by a Nvidia (Santa Clara, California, United States) quadro k600 graphics card. The monitor was calibrated by generating a gamma-corrected lookup table (LUT) converting XYZ to RGB, accounting for output nonlinearities, based on the monitor primaries recorded with a Konica Minolta (Osaka, Japan) CS-2000 spectroradiometer. Observers sat approximately 60 cm from the monitor and viewed the screen binocularly with free head movement. At this distance, the screen subtended 41 × 23 degrees of visual angle. The computer presenting stimuli was changed after the first 10 observers as the original PC malfunctioned. The monitor was recalibrated following this change.

#### Stimulus generation

Three unique scenes were pregenerated, each having a different arrangement of six different three-dimensional shapes. Each shape in each scene had a different surface reflectance, except the central shape, which kept the same surface reflectance in each scene. For each scene, two versions were generated—one glossy and one matte. Each of these scenes was shown under five different illuminations (neutral, red, green, blue, and yellow). This resulted in a total of 30 unique stimuli.

The three-dimensional scenes were created using Blender (https://www.blender.org/) and rendered using Mitsuba (http://www.mitsuba-renderer.org/), compiled for spectral rendering with 30 spectral bands. They were then scaled, such that each scene had a mean luminance of 60 cd/m${}^{2}$, which ensured most pixels were in gamut. The scenes were tone mapped by clipping any out of gamut values (negative RGB values were clipped to 0 and RGB values greater than 1 were clipped to 1), to prevent saturation at high luminance levels. The pixels which were clipped in each glossy scene, are shown in the Supplementary Figure S2. Most were on green/blue shapes, and none of the pixels making up the specular highlights were truncated. Clipping did not significantly shift the chromaticities, as can be seen in the Supplementary Material, which shows the mean scene chromaticities against the illumination chromaticities. Finally, the scenes were converted to RGB images using the LUT constructed from the calibration described above.

**Figure 3.**
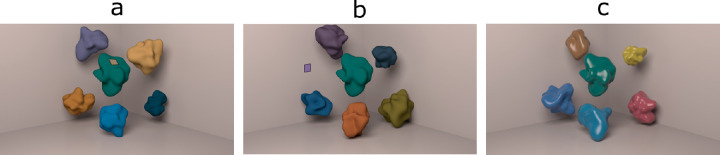
Example of matte scenes containing matching patch on shape (a) and wall (b). Example of a glossy scene (c).

The scenes consisted of six shapes sitting in a box with gray walls (see Figure 3). The background was the brightest surface in the scene with a flat spectral reflectance of 0.8. This ensured the background could be used for the “brightest is white” cue, even when specular highlights were present. The specularity of the background was 0.5 and the alpha (a measure of roughness) was 0.05—slightly rough. A square area light, the same size as each wall of the room, was positioned just behind and above the camera, angled toward the junction of the two vertical walls. This produced a diffuse light across the scene.

Fourteen unique naturalistic-looking “blobby shapes” were generated for the scenes using ShapeToolbox (Saarela, 2018). Each shape was created using a sphere base shape with five sets of 20 randomly positioned bumps. For each set of bumps, the amplitude was randomly selected with an upper limit of 0.5 radians and a *SD* between 0.2 and 0.3. In addition, each of the 14 shapes were modulated using four sinusoidal modulations with a random frequency below 10 cycles/2π radians and an amplitude between 0.05 and 0.2 radians. One of these shapes was selected to be the standard shape, which was always in the center of the room, and had a surface reflectance of Munsell chip 5BG4/6. This shape subtended approximately 8 degrees of visual angle. The reflectance functions of each Munsell chip used were retrieved from https://www.uef.fi/web/spectral/munsell-colors-matt-spectrofotometer-measured. The standard shape's reflectance was consistent over all stimuli to reduce any noise between trials, and the shape was selected to have a flat surface for the matching patch to sit on.

Three unique scenes were generated from combinations of the 14 unique shapes. Scene acted as a random variable, as we were interested in effects common to different shape, surface reflectance, and location combinations. In each scene, the five shapes other than the standard shape were taken randomly from the set of 13 remaining shapes and randomly rotated so that a different side was viewed in each scene. The shapes were positioned randomly within a region which ensured no overlap so all the shapes were visible.

For each of the three scenes, two versions were created using the same arrangement of shapes—one glossy and one matte. Within each pair, the corresponding glossy and matte shapes had the same diffuse surface reflectance but different levels of specular reflectance. Despite the presence of specular highlights on the glossy shapes, the mean chromaticity across scenes did not vary much between matte and glossy scenes (see Supplementary Figure S1 for details). Both matte and glossy materials were created using the plastic material in Mitsuba. The roughness (alpha) of both materials was set to 0.1, which is relatively rough. This roughness meant that the specular highlights on the glossy shapes were not too bright to be presented on the monitor. For the matte material, the specular reflectance was 0, meaning they were perfectly matte and contained no specular highlights. The glossy material had a specular reflectance of 1, which ensured the shapes had specular highlights. The surface reflectance of the six shapes within each scene was chosen such that the average surface reflectance was neutral (not differing by more than 1ΔE${}_{u^{*}v^{*}}$ from a flat surface reflectance). Each of the three scenes contained a different set of six surface reflectances to ensure any findings were not specific to the stimuli used. The Munsell chips used in each of the three arrangements are given in Table A.1.

**Figure 4.**
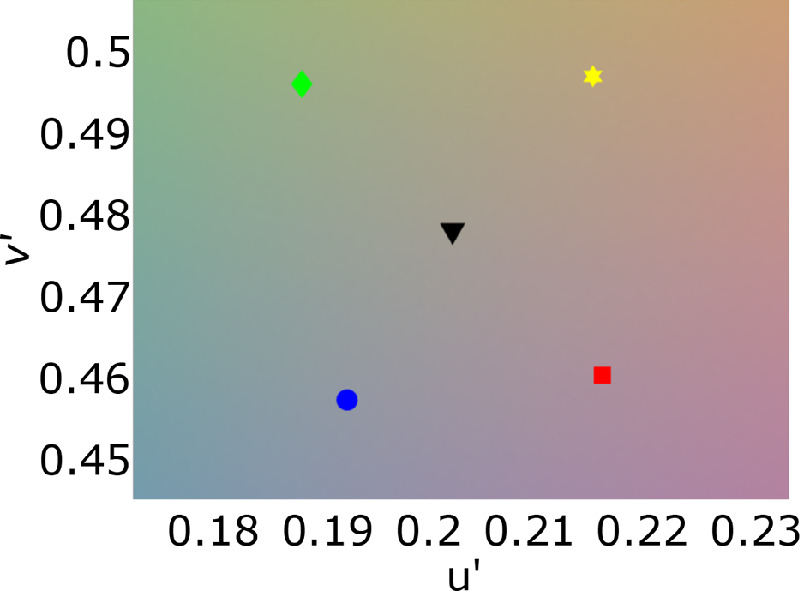
Illumination chromaticities in L′u′v′ space.

The illuminations selected comprised a neutral D57 (CCT 5698), which has been proposed to be the mean of all daylights (Nascimento, Amano, & Foster, 2016), and four other illuminations, all 30ΔE${}_{u^{*}v^{*}}$ away from neutral (using the neutral illumination as the white point). These illuminations included two along the daylight axis in the blue and yellow directions and two illuminations with chromaticities perpendicular to the daylight axis at CCT 5698K in CIE L′u′v′ space. These illuminations will hereafter be referred to as neutral, blue, yellow, red, and green, respectively. See Table 1 and Figure 4 for chromaticity coordinates of the illuminations used in CIE Yxy and CIE L′u′v′ color space.

**Figure 5.**
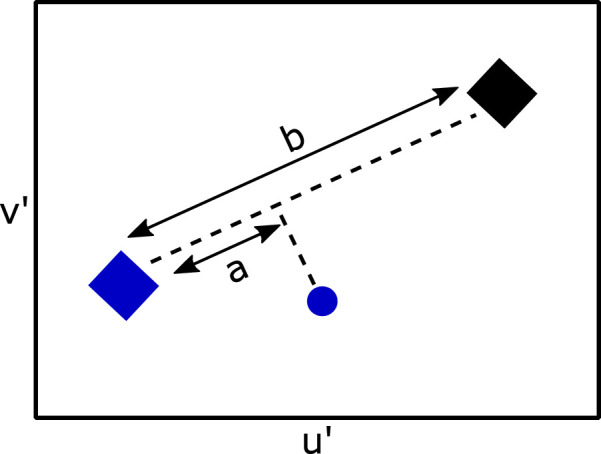
Example settings for calculating a CCI. The blue circle is $e_{i}$, the blue diamond is $t_{i}$, and the black diamond is $r_{i}$. See text for explanation of abbreviations.

**Table 1.** Coordinates of the illuminations used in CIEYxy and CIEL'u′v′ color spaces.

Illumination	x	y	u′	v′
Neutral (D57)	0.328	0.344	0.203	0.478
Blue	0.297	0.314	0.193	0.458
Yellow	0.364	0.372	0.216	0.497
Red	0.329	0.310	0.217	0.460
Green	0.327	0.382	0.189	0.496

Both matte and glossy versions of the scenes were rendered under all five illuminants for a total of 10 scenes, each with three different arrangements of shapes and reflectances.

#### Task

We used an achromatic adjustment task in these experiments, as this has been used extensively to measure color constancy (Werner, 2007; Boyaci, Doerschner, & Maloney, 2004; Yang & Maloney, 2001; Delahunt & Brainard, 2004; Brainard, 1998), and Speigle and Brainard (1999) have shown that results from achromatic adjustment agree with those from asymmetric matching. Observers were shown a scene with a square patch (approximately 1.4 degrees of visual angle) either on the central shape in the scene (see Figure 3a) or on one of the background walls (Figure 3b). The starting chromaticity of the patch was chosen randomly from an area within 38 $\Delta E_{u^{*}v^{*}}$ from neutral (D57). The luminance of the matching patch was fixed at 60 cd/m${}^{2}$, to match the mean luminance of the scenes. Observers adjusted the color of this patch until it appeared gray, using buttons on an Xbox controller, which altered the CIE u*v* color coordinates. There were three different step sizes such that observers could shift the chromaticity by 10 (big), 5 (medium), or 1 (small) ΔE in the u* or v* directions. Observers were free to choose which step sizes to use. The instructions given to observers were adapted from Radonjić and Brainard (2016), which emphasized considerations of reflectance properties, and are thus aimed to optimize color constancy. These are given in the Appendix.

#### Procedure

Each of the illumination-specularity combinations was presented nine times within a session, for a total of 90 trials per session. This consisted of three repeats of each of the three unique scenes. Half the observers completed the first session with the patch on the wall followed by the second session with the patch on the shape; the other half did the sessions in reverse order. Each session lasted approximately 1 hr. The two sessions were separated by a gap ranging from 1 day to 22 days between observers.

Observers were first dark adapted for 2 min. They were then given five practise trials in which they adjusted the color of a circular patch on a black background to appear gray, green, blue, yellow, and red. This was to ensure observers understood how the controllers worked. They were then presented with an empty scene, consisting of the box with gray walls and floor but no shapes, under one of the five illuminants. The order of the illumination colors was randomized between observers. Following 2 min of adaptation to an empty room, observers made achromatic settings for the 18 trials under this illumination color (9 glossy; 9 matte). For each illumination, the nine matte scenes were presented followed by the nine glossy scenes or vice versa. This order was randomized for each illumination and observer. After completing all trials under one illuminant, observers were adapted to the next illumination for 2 min before completing all trials under that illuminant, until all conditions had been completed. Although 2 min is not sufficient for complete global adaptation, each illumination condition lasted approximately 10 min following adaptation, meaning most achromatic settings were made after at least 5 min of adaptation. By this time, global adaptation would have stabilized.

#### Data analysis

For all data analysis, settings were converted from L*u*v* to L′u′v′. Both color spaces are designed to be perceptually uniform (that is, any two points at a fixed distance from each other will theoretically be approximately equally discriminable anywhere in the color space), but u* and v* vary with changes in L*, whereas u′ and v′ remain constant for any value of L′ (Wyszecki & Stiles, 1982). Therefore, conducting analysis in L′u′v′ ensures that the chromaticity does not depend on luminance.

##### Exclusion criterion

In 95% of trials pooled across observers, at least four adjustments (button presses) were made to the patch color before a setting was submitted as gray. We called any trial with fewer than four adjustments an outlier and removed it from the analysis. After exclusion, there remained at least seven valid trials for each observer and condition.

##### Variable error

A two-dimensional Gaussian was fit to the settings made for each condition and for each observer separately. This was done by calculating a covariance matrix of the data and calculating the eigenvalues and eigenvectors of this matrix. To calculate a single measure of variable error, the area of an ellipse encompassing the eigenvectors of this Gaussian was calculated:

(1) $$Area=\pi \times \sqrt{Eigenvalue_{1}}\times \sqrt{Eigenvalue_{2}}$$

As these Gaussians were fit to only nine settings, they could have resulted in poor fits. Therefore, in addition, the standard deviation of the settings was calculated separately in the u′ and v′ dimensions. This additional measure also allowed us to further explore any significant effects found for the overall variable error.

##### Color constancy index (CCI)

Color constancy indices were calculated using the equivalent illumination method of Brainard (1998), which has been used in many studies of color constancy (Yang & Maloney, 2001; Delahunt & Brainard, 2004; Kraft, Maloney, & Brainard, 2002). The equivalent illumination method effectively recenters an individual observer's settings on their individual settings under the neutral reference illumination. This takes into account the fact that the observers’ internal representations of gray may not be spectrally uniform and predicts what chromaticity observers’ setting would have been if they did have a spectrally uniform representation of grey. These were calculated relative to the settings made under the neutral illumination as follows. All settings and illumination chromaticities were first transformed to LMS space. The reference illumination ($r_{i}$) was defined as the cone coordinates of the neutral illumination. The reference match ($r_{m}$) was the cone coordinates of the mean setting made under the neutral illumination. The test illumination ($t_{i}$) was defined as the cone coordinates of the chromatic illumination for this condition (blue, green, red, or yellow). The test match ($t_{m}$) was the mean cone coordinates of the settings made under this condition. The equivalent illumination ($e_{i}$) was then calculated as

(2) $$\begin{array}{c} e_{i}=r_{i}\frac{t_{m}}{r_{m}},\; \end{array}$$

for each cone class (L, M, and S) separately.

The equivalent illumination was then converted to CIE L′u′v′ in order to calculate a color constancy index, CCI, as

(3) $$\begin{array}{c} CCI=1-\frac{a}{b},\; \end{array}$$

where a is the length of the vector projection of $e_{i}-t_{i}$ onto $r_{i}-t_{i}$ and b is the length of $r_{i}-t_{i}$ (Figure 5).

### Results

**Figure 6.**
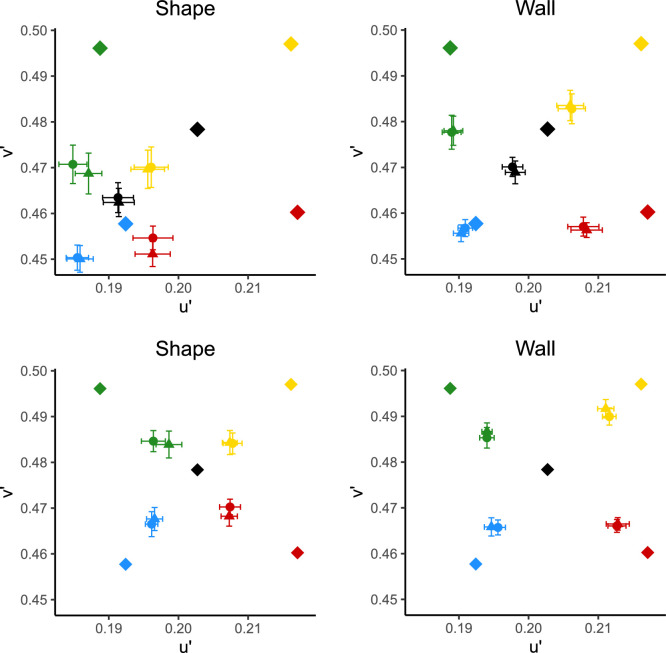
Top: raw achromatic settings in u′v′. Bottom: equivalent illuminants in u′v′. Diamonds represent illuminant chromaticities; circles represent mean settings made in glossy scenes, and triangles represent mean settings made in matte scenes. Colors represent illuminant colors. Error bars show ± 1 standard error of the mean.

The average achromatic settings over all observers and repeats is shown in Figure 6 (top row), with error bars representing ± 1 standard error of the mean. As can be seen, settings are all drawn strongly toward the blue direction of u′v′ color space, with the mean setting under neutral, when the patch was on the shape, being almost on the blue illumination's chromaticity. Therefore, for clarity, the equivalent illuminants (described above) are shown on the bottom row of Figure 6. The mean color constancy index across all observers and conditions was 0.519 ± 0.0240.

**Figure 7.**
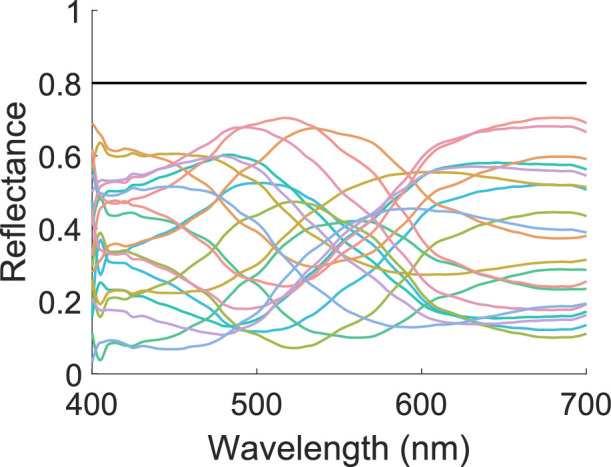
Spectral reflectance functions of 21 patches used in Mondrian. The 20 colored lines are the original and inverted Munsell chip reflectances. The black line is the reflectance of the white patches.

To test whether people were using specular highlights as a cue to improve their color constancy and decrease variability, we ran a 4 (illumination) × 2 (material) × 2 (position) analysis of variance (ANOVA) on the CCIs and a 5 (illumination) × 2 (material) × 2 (position) ANOVA on the variable error. The results are shown in Tables 2 and 3, respectively.

**Table 2.** Results from ANOVA on CCIs.

Effect	*F* (*df*)	*p*	$\eta_{p}^{2}$
Illumination (main)	2.156 (1.575, 20.472)	.149	.142
**Position (main)**	**8.361 (1, 13)**	.013^*^	**.391**
Material (main)	.067 (1, 13)	.799	.005
Illumination × Material	.375 (3, 39)	.771	.028
Illumination × Position	1.265 (3, 39)	.300	.089
Material × Position	2.053 (1, 13)	.175	.136
Illumination × Material × Position	1.553 (3, 39)	.216	.107

^*^Indicates significance at the p < 0.05 level.

**Table 3.** Results from ANOVA on variable error (area of ellipse fit to two-dimensional Gaussian).

Effect	*F* (*df*)	*p*	$\eta_{p}^{2}$
Illumination (main)	2.409 (4, 52)	.061	.156
**Position (main)**	**55.965 (1, 13)**	<.001^***^	**.811**
Material (main)	1.444 (1, 13)	.251	.100
Illumination × Material	1.050 (4, 52)	.390	.075
Illumination × Position	.267 (4, 52)	.898	.020
Material × Position	.295 (1, 13)	.596	.022
Illumination × Material × Position	1.522 (4, 52)	.210	.105

^***^Indicates significance at the p < 0.001 level.

There was no evidence for a main effect of specular highlights on either CCI or variable error, against our first hypothesis. In addition, there were no interactions of specular highlights with illumination or position, thereby not supporting Hypothesis 2. Against our third hypothesis, there was no main effect of illumination on CCI or any significant interactions.

We found a significant main effect of patch position on both CCI and variable error. When the patch was on the wall, there was a significantly higher CCI (mean = .619, *SD* = .305) than when the patch was on the shape (mean = .418, *SD* = .383). Similarly, there was a smaller variable error when making adjustments to the patch on the wall (mean = $6.24\times 10^{-5}$, *SD* = $4.31\times 10^{-5}$) than on the shape (mean = $1.24\times 10^{-4}$, *SD* = $7.26\times 10^{-5}$), suggesting the estimates were more reliable when made on the wall than on the shape. The effect on variable error was further investigated by looking at the error made in u′ and v′ separately. For u′, there was a significant main effect of patch position $\left(F\left(1,13\right)=36.501,p<0.001,\eta_{p}^{2}=.737\right)$, with a higher variable error on the shape (mean = $.564\times 10^{-3}$, *SD* = $.200\times 10^{-3}$) than on the wall (mean = $.381\times 10^{-3}$, *SD* = $1.69\times 10^{-3}$). Similarly, for v′ there was a significant main effect of position $\left(F\left(1,13\right)=15.513,p=0.002,\eta_{p}^{2}=.544\right)$, with a higher variable error on the shape (mean = $7.62\times 10^{-3}$, *SD* = $2.87\times 10^{-3}$) than on the wall (mean = $5.84\times 10^{-3}$, *SD* = $2.74\times 10^{-3}$). The difference between settings on wall and shape suggested that the effects of simultaneous contrast between the patch and its local surround influence observers’ settings.

### 2.3 Interim discussion

In this experiment, we tested 14 observers, which is more than have been used in much of the previous research into color constancy: five naive in Yang and Maloney (2001), eight in Experiment 1 of Lee and Smithson (2016), and four in Experiment 2. This allowed us to conduct inferential statistical tests. Overall, the mean CCI was slightly lower than other studies measuring color constancy in three-dimensional rendered scenes (e.g., Yang & Maloney [2001] found average CCIs of 0.65; Delahunt and Brainard [2004] found CCIs ranging from 0.67 to 0.81 when cues were consistent and valid).

Against our hypotheses, there were no main effects of specular highlights on either CCIs or variable error, or any significant interactions. It should be noted, however, that in these scenes, there was a strong cue from the uniform background, which reflected the chromaticity of the illumination. This strong cue may have hidden any smaller effects of specular highlights, which were predicted to improve color constancy. This is in agreement with findings from Xiao et al. (2012), who found specular highlights only improved color constancy when a local surround cue was silenced.

It was also predicted that observers would show a higher degree of color constancy for scenes illuminated by daylights (blue and yellow) than nondaylights. Although there was no effect of illumination on CCI, there was a noticeable bias toward blue for the raw settings, consistent with previous studies (Winkler, Spillmann, Werner, & Webster, 2015; Weiss et al., 2017). This could reflect observers, internal representations of gray being bluish (i.e., containing relatively more short wavelengths) rather than spectrally neutral. Alternatively, it may result from having a prior for bluish illuminations (Pearce et al., 2014; Delahunt & Brainard, 2004), which causes observers to attribute bluish components of reflected light to the illumination rather than surface reflectance and thus perceive bluish surfaces as neutral (see Aston & Hurlbert, 2017).

A noticeable finding from this experiment was that observers performed significantly better (in terms of both CCI and variable error) when the patch was on the back wall than on the turquoise shape. In fact, when the patch was on the back wall, CCIs were close to those of previous studies, many of which had the matching patch on a back wall. One possible explanation for this difference is the difference in lightness and chromatic contrast. When the matching patch was on the back wall, it had a luminance slightly lower than that of the local surround, which ranged from 70.31 to 70.32 cd/m${}^{2}$ across different illuminations. However, on the turquoise shape, the patch had a much higher luminance than the surround, which had a mean luminance ranging from 20.73 to 22.34 cd/m${}^{2}$ across the different illumination conditions. Using a matching patch with a higher luminance than the local surround has been shown to decrease color constancy (e.g., Bäuml, 2001; Helson, 1938), and having greater luminance contrast between matching patch and surround (in either direction) results in poorer color constancy (Werner & Walraven, 1982; Kuriki, 2006). Similarly, chromatic contrast has been found to affect appearance (Shevell & Wei, 1998; Werner, 2014; Faul, Ekroll, & Wendt, 2008), which could explain the difference in performance across patch positions. While the black border between matching patch and surround will have decreased the effect of simultaneous contrast (Faul et al., 2008; Blackwell & Buchsbaum, 1988), it was not sufficient to eliminate the effect. An alternative explanation for this difference is that the uniform gray background is giving strong contributions of global and local adaptation to constancy, thus masking any subtler effects of the specular highlights.

## Experiment 2

In Experiment 2, we tested whether we would see an effect of specular highlights when the uniform background was replaced with a checkerboard, Mondrian-like background. This was designed to prevent use of a uniform background color as a direct reference cue, to limit the contribution from local surround adaptation on the wall, and to more closely equate the local surround across patch positions. The average chromaticity of the wall's reflectance remained neutral so observers could theoretically still use the global mean cue.

### Methods

#### Observers

A different group of 14 naive psychology undergraduates participated in this experiment to earn course credits. They had a mean age of 20.93, ranging from 18 to 41, and included 11 females. All observers were screened for color blindness using Ishihara plates and had normal or corrected-to-normal visual acuity.

#### Materials and apparatus

The materials and apparatus were the same as in Experiment 1.

#### Stimulus generation

**Figure 8.**
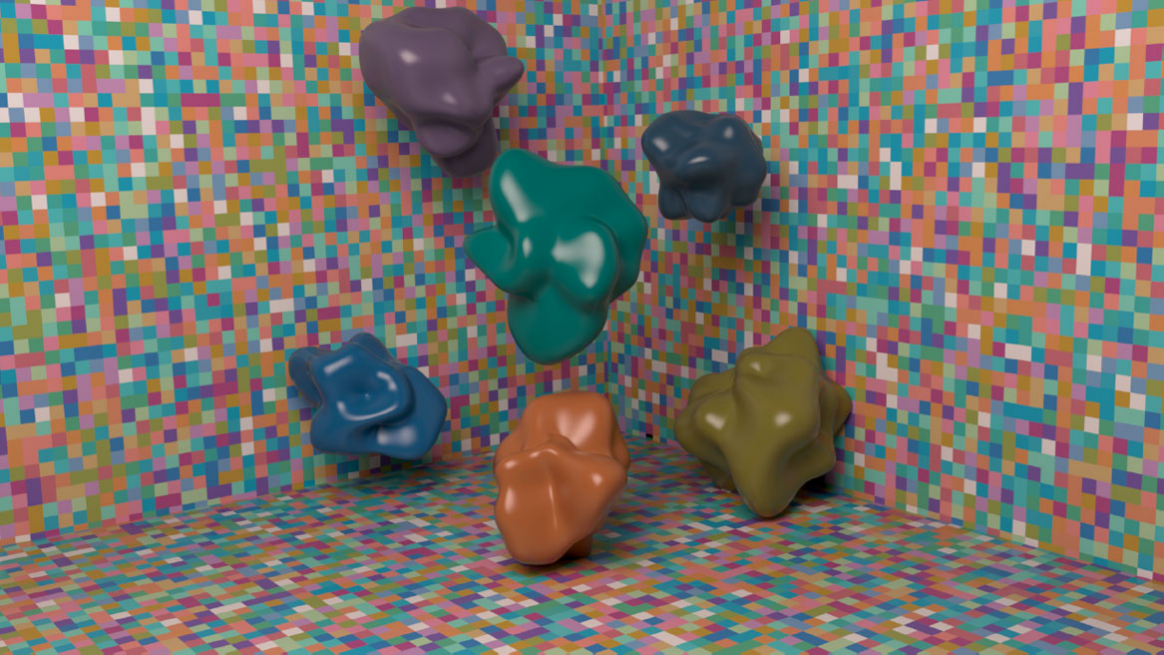
Example scene.

The stimuli were the same as in Experiment 1 except the background wall consisted of a checkerboard pattern with multiple instances of 20 distinct surface reflectances (henceforth, Mondrian background). To generate this, 10 Munsell chips were selected (5B7/8, 5BG7/8, 5G7/8, 5GY7/8, 5Y7/8, 5YR7/8, 5R7/8, 5RP7/8, 5P7/8, and 5PB7/8). These were reconstructed using the basis functions of Parkkinen, Hallikainen, and Jaaskelainen (1989). To create the remaining 10 surfaces, the weights on the basis functions were inverted, and the spectra shifted to have the same mean reflectance as the original surface. As a result, the average of the 20 surfaces has a flat spectral reflectance. In addition to these 20 reflectances, a spectrally nonselective surface was also used, which reflected 80% of light at all wavelengths, which is more light than any of the other surfaces. This ensured the brightest-is-white cue was still valid. The reflectance spectra of all 21 surfaces are shown in Figure 7. Each wall and floor consisted of a 40×40 array of small planes (varying from 0.4 degrees at the furthest point to 0.6 degrees of visual angle at the nearest point), each randomly assigned one of the reflectances described above. An example scene is shown in Figure 8.

The rendered scenes were scaled to have a mean luminance of 40 cd/m${}^{2}$—lower than in Experiment 1. This was to keep the luminance of the shapes similar to Experiment 1, despite the darker background.

The rendered adaptation rooms were the same as in Experiment 1, with blank neutral walls. They were scaled to have a mean luminance of 40 cd/m${}^{2}$ to match the experimental stimuli used.

#### Task

The task was to set a patch to appear gray, on either the central shape or the back wall, as in Experiment 1. In order to overcome the difference in local lightness contrast in Experiment 1, in which the matching patch was lighter than the surround on the shape but darker than the surround on the wall, in the present experiment, the luminance of the matching patch was set to 20 cd/m${}^{2}$. The luminance of the local surround on the shape ranged from 20.06 to 21.54 cd/m${}^{2}$ and on the wall ranged from 41.80 to 42.15 across the different illumination conditions. Therefore, decreasing the luminance of the matching patch ensures it is close to, or lower than, the local surround in all conditions.

#### Procedure

The procedure for each session was identical to Experiment 1, and the order of the sessions was counterbalanced between observers. However, this time, observers were given the option to do both sessions in immediate succession, and 11 observers chose to do this. For the remaining 3 observers, the sessions were completed between 7 and 19 days apart. Each session lasted approximately 1 hr.

#### Data analysis

The data were analyzed in the same way as in Experiment 1. Using the same exclusion criterion, only three trials were included for one observer under one condition. All other conditions had at least five valid trials.

#### Modeling local surround

In further analyses, we wished to compare observers’ matches with those predicted from the local adaptation cue alone. We modeled the predicted CCIs which would be obtained using only local surround information, as follows. The local surround for the patch on the wall was defined as the area encompassing at least one square of Mondrian in every direction. On the shape, it was defined as an area the same size as the wall's local surround, minus any pixels making up the Mondrian. This local surround subtended approximately 0.5 degrees of visual angle on each side of the matching patch. The mean chromaticity of the local surround was calculated from the hyperspectral rendering for one matte scene under each of the five illuminants. It should be noted that the local surround did not extend onto any highlights, meaning there was no difference in the local surround on matte compared to glossy shapes. A CCI was calculated for each patch position under each illuminant according to the equivalent illumination calculation described in Equations 2 and 3. $t_{m}$ was defined as the chromaticity of the local surround under the chromatic illuminants, and $r_{m}$ was defined as the chromaticity of the local surround under the neutral illuminant. $t_{i}$ and $r_{i}$ were defined as in Equation 2. Note that the equivalent illumination calculation ensures the overall shift away from neutral in local surround on the shape does not impair predicted CCIs.

### Results

**Figure 9.**
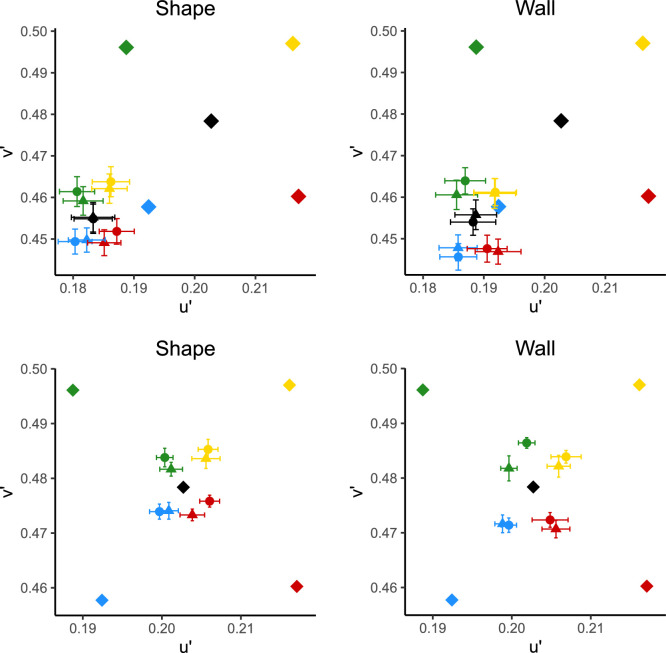
Top: raw settings in u′v′, Bottom: equivalent illuminants in u′v′. Diamonds represent illuminant chromaticities; circles represent mean settings made in glossy scenes and triangles represent mean settings made in matte scenes. Colors represent illuminant colors. Error bars show ± 1 standard error of the mean.

The raw achromatic settings averaged across all 14 observers are shown in Figure 9 (top row). As in Experiment 1, and in line with some previous research (Weiss et al., 2017), there is a clear bias in the settings toward a blue daylight illumination. However, there is a stronger bias toward blue when the patch was on the back wall than in Experiment 1. The equivalent illuminants are plotted in Figure 9 (bottom row) for clarity. The mean CCI over all observers and conditions was 0.255 ± 0.0160.

The results of the 4 (illumination) × 2 (position) × 2 (material) ANOVA on CCIs and 5 (illumination) × 2 (position) × 2 (material) ANOVA on variable error are shown in Tables 4 and 5, respectively. In line with our first hypothesis, we found a significant main effect of material on CCI, with a higher CCI for scenes containing glossy shapes (mean = .272, *SD* = .220) compared to matte shapes (mean = .239, *SD* = .237). This effect was not found on variable error.

**Table 4.** Results of ANOVA on CCIs.

Effect	*F* (*df*)	*p*	$\eta_{p}^{2}$
Illumination (main)	.302 (3, 39)	.823	.023
**Position (main)**	**5.469 (1, 13)**	.036^*^	**.296**
**Material (main)**	**5.958 (1, 13)**	.030^*^	**.314**
Illumination × Material	1.863 (2.097, 27.258)	.173	.125
Illumination × Position	.773 (3, 39)	.516	.056
Material × Position	.526 (1, 13)	.481	.039
Illumination × Material × Position	.091 (2.002, 26.023)	.914	.007

^*^Indicates significance at the p < 0.05 level.

**Table 5.** Results of ANOVA on variable error (area of ellipse fit to two-dimensional Gaussian).

Effect	*F* (*df*)	*p*	$\eta_{p}^{2}$
Illumination (main)	1.188 (4, 52)	.327	.084
**Position (main)**	**7.219 (1, 13)**	.019^*^	**.357**
Material (main)	2.374 (1, 13)	.147	.154
Illumination × Material	1.364 (4, 52)	.259	.095
Illumination × Position	.637 (4, 52)	.638	.047
**Material × Position**	**7.531 (1, 13)**	.017^*^	**.367**
Illumination × Material × Position	.270 (4, 52)	.896	.020

^*^Indicates significance at the p < 0.05 level.

**Figure 10.**
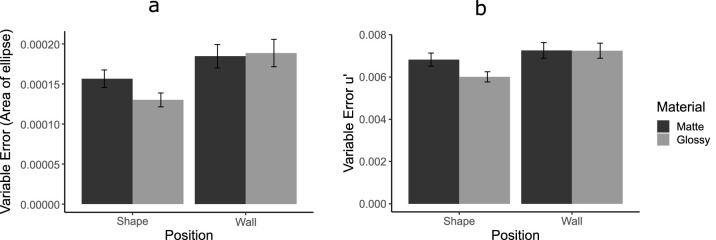
(a) Variable error (area of an ellipse fit to eigenvectors of Gaussian) when the matching patch was on the shape (left) and wall (right). Dark gray bars are for scenes containing matte shapes; light gray bars are for glossy shapes. Error bars are ± 1 standard error of the mean. (b) The same interaction for variable error in u′, measured as the standard deviation across all settings.

In support of our second hypothesis, we also found a significant interaction between material and position on variable error. This interaction is shown in Figure 10a. To further explore the interaction, paired samples *t* tests were conducted separately for patch on shape and patch on wall conditions. When the patch was on the shape, but not on the wall, there was a significant effect of material (*t*(13) = 2.376, *p* = 0.034), with less variability of estimates for scenes containing glossy shapes (mean = $1.30\times 10^{-4}$, *SD* = $4.5\times 10^{-5}$) than for scenes containing matte shapes (mean = $1.56\times 10^{-4}$, *SD* = $6.0\times 10^{-5}$). To determine whether this effect was driven by variability in u′, v′, or both, we ran further ANOVAs on the standard deviation in both directions separately. There was a significant Material × Position interaction in u′ $(F\left(1,13\right)=4.651,p=0.050,\eta_{p}^{2}$= .264) but not in v′. The interaction in u′ is shown in Figure 10b, and is driven by significantly less variable error in scenes containing glossy shapes (mean = .0060, *SD* = .00143) than in scenes containing matte shapes (mean = .0068, *SD* = .00180), when the patch is on the shape: *t*(13) = 3.106, *p* = 0.008. There was no significant difference in variable error in u′ between scenes containing matte and glossy shapes when the patch was on the back wall.

Against our third hypothesis, there was no significant main effect of illumination on CCI, or any significant interactions involving illumination.

**Figure 11.**
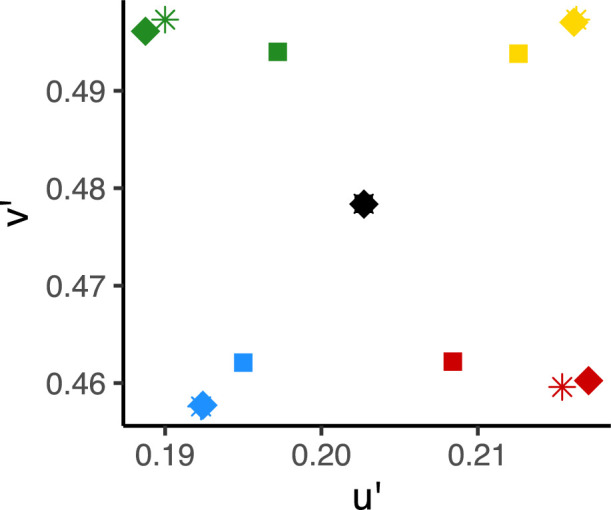
Predicted optimal performance using local surround information only. As in previous figures, diamonds indicate actual illumination chromaticities. Squares reflect optimal performance when making estimates on the central shape; asterisks are optimal performance when making estimates on the back wall (using local surround information only).

As in Experiment 1, a significant main effect of position was found on CCIs, with significantly higher CCIs when the patch was on the back wall (mean = .286, *SD* = .253) than on the shape (mean = .225, *SD* = .222). To investigate this further, we modeled the predicted CCIs for each patch position, using local surround information only, as described above. The predicted equivalent illuminants from this analysis are plotted in Figure 11. As expected, there was a greater bias in the local surround when the patch was on the turquoise shape than on the back wall. It should be noted, however, that due to the random nature of the Mondrian background, the local surround on the wall was slightly biased toward blue. Modeling optimal performance using only local surround information, higher CCIs are predicted when the patch is on the back wall (mean = 0.980 across all illuminations) than on the shape (mean = 0.744 over all illuminations).

In addition, there was a significant main effect of position on variable error, with significantly more variable error when the patch was on the back wall (mean = $1.866\times 10^{-4}$, *SD* = $1.326\times 10^{-4}$) than on the shape (mean = $1.433\times 10^{-4}$, *SD* = $8.347\times 10^{-5}$). To further explore this, we looked at the ANOVA conducted on variable error in u′ and v′ separately. There was a significant main effect of position on variable error in u′ $\left(F\left(1,13\right)=10.009,p=0.007,\eta_{p}^{2}=.435\right)$, but not v′ (*p* = 0.093). In u′, there was significantly more variable error when the patch was on the wall (mean = $7.251\times 10^{-3}$, *SD* = $3.040\times 10^{-3}$) than on the shape (mean = $6.416\times 10^{-3}$, *SD* = $2.340\times 10^{-3}$).

### Interim discussion

In the present experiment, unlike Experiment 1, we did find a significant main effect of material on CCIs, supporting the first hypothesis that the presence of specular highlights can improve color constancy. In addition, there was a decrease in variability of estimates for scenes containing specular highlights when observers were making illumination estimates on the shapes but not on the back wall, in terms of both overall variability and standard deviation in u′. This is in agreement with Yang and Maloney (2001), who found no effect of perturbing specular highlights when judgments were made on a back wall. Taken together, these findings suggest that observers are able to make use of specular highlights when estimating illumination chromaticities but segment the scene into different illumination frameworks before making estimates. An alternative explanation is that observers may be relying on specular highlights more when making estimates on the shape because the local surround cue is harder to use so more weight is applied to the highlights. With the Mondrian background, observers appear to rely more on the specular highlights than when scenes contained a uniform neutral background (Experiment 1). It is important to note that CCIs were, on average, much lower here than in Experiment 1. This could be due to the Mondrian background making the local and global mean cue harder to use. Nevertheless, the fact that an effect of specular highlights was found here suggests that the high overall performance in Experiment 1 may have masked any potential effect of highlights.

As in Experiment 1, we did not find the predicted benefit for scenes illuminated by daylight illuminants. There was still a strong bias toward blue in the raw settings, but when converted to equivalent illuminants, there was no difference in constancy between the illuminations. This suggests observers' internal representations of gray are not spectrally uniform but biased toward spectra containing more shorter wavelengths, which appear bluish-gray. However, an alternative explanation—at least when the matching patch was on the shape—is that the turquoise local surround from the shape could have biased settings toward blue.

## Experiment 3

In Experiment 3, we aimed to test to what extent the bias in local surround could have driven a difference between performance on the wall versus on the shape in Experiments 1 and 2. To this end, we varied the reflectance of all shapes, including the one containing the matching patch, on every trial. This allowed us to determine whether the effect of position depended on having the same local surround on the shape on every trial. This also ensures any findings are not specific to the limited range of stimuli used in Experiments 1 and 2. However, changing the color of the shape on a trial-by-trial basis is likely to add variability and noise to estimates. In order to counteract this, we ensured observers used the smallest adjustment step size, thus making more finely tuned settings overall than in the previous experiments.

### Methods

#### Observers

Fourteen naive paid volunteers participated in this study. They had a mean age of 24.79, ranging from 20 to 35, and included eight females. All observers were screened for color blindness using Ishihara plates and had normal or corrected-to-normal vision.

#### Materials and apparatus

The materials and apparatus were the same as in Experiment 2.

#### Stimulus generation

The same Mondrian room from Experiment 2 was used in the present experiment. Rather than having three scenes, we selected one scene from Experiment 2 (using the reflectances in Set 1 in Table A.1). This meant all stimuli had identical shapes. For each trial and each observer, the six reflectances were randomly assigned to each of the six shapes. This meant that the scenes were more variable, and the shape containing the matching patch could have one of six different reflectances. It is important to note that the average chromaticity of all six reflectances under all illuminants used did not differ by more than 1ΔE${}_{L^{*}u^{*}v^{*}}$ from a neutral surface with the same mean luminance. This means that, over all trials, on average, there should be no biased local surround when the patch is on the central shape.

#### Task

The task was the same as in Experiment 2. However, in order to decrease the noise in observers' settings, they were now required to use the smallest adjustment step size (1ΔE) at least once before a match could be recorded.

#### Procedure

There were nine repeats under each condition, with a different arrangement of reflectances on each repeat, individually defined for each observer.

Initially, observers completed a session with the patch on a wall and a session with the patch on the shape, as in Experiments 1 and 2, spread out between 1 and 22 days. However, when running the wall condition, some data were lost for 11 observers due to computer error. To rectify this, a third session was conducted in which observers made settings on both the wall and the shape, with the position changing halfway through. This was conducted at a later date, between 49 and 82 days after the latter of the first two sessions. For this, half the trials from the first session and half the trials from the second session were used. The order was matched to the original sessions such that if an observer participated in the shape session before the wall session, in the final session they would see the shape trials first. Although this was conducted to replace missing data, it also allowed us to test for consistency in achromatic settings over time.

No data were lost for the remaining three observers, so they just took part in two sessions each.

#### Data analysis

The same exclusion criterion was applied as in Experiments 1 and 2, and CCI and variable error were calculated in the same way. After exclusion, all conditions had at least eight valid trials.

The data collected on the shape session and shape half of the third session were compared to test for a difference across time points. To determine whether there was a difference between the sessions, a 2 (session) × 4 (illuminant) ANOVA was conducted on the CCIs, collapsed across both material types as only one material was used under each illuminant in the third session. There was no significant main effect of session, illuminant, or interaction. From this, it was concluded that observers' illumination estimates are consistent over time.

For the full analysis below, data collected on the shape session were analyzed as in Experiments 1 and 2. For the 11 observers who completed three sessions, the data from the wall session and the wall half of the final session were combined for analysis. The data from the remaining three observers were analyzed as normal.

### Results

**Figure 12.**
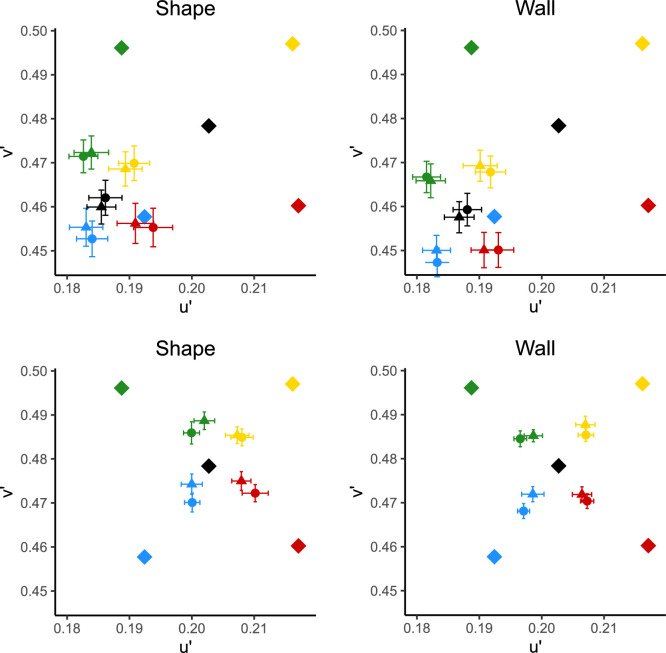
Top: raw settings in u′v′. Bottom: equivalent illuminants in u′v′. Diamonds represent illuminant chromaticities; circles represent mean settings made in glossy scenes, and triangles represent mean settings made in matte scenes. Colors represent illuminant colors. Error bars show ± 1 standard error of the mean.

The mean raw settings over all observers and repeats are shown in Figure 12 (top row). As can be seen, there is still a strong pull toward blue in these settings in both the wall and shape conditions. The equivalent illuminants are shown for clarity in Figure 12 (bottom row). The mean CCI over all conditions and observers was 0.361 ± 0.0193—higher than in Experiment 2 but lower than in Experiment 1.

As before, a 4 (illumination) × 2 (position) × 2 (material) ANOVA was run on CCIs, and a 5 (illumination) × 2 (position) × 2 (material) ANOVA was run on the variable error. The results of these are shown in Tables 6 and 7, respectively.

**Table 6.** Results of ANOVA on CCIs.

Effect	*F* (*df*)	*p*	$\eta_{p}^{2}$
Illumination (main)	.119 (3, 39)	.948	.009
**Position (main)**	**4.654 (1, 13)**	.050^*^	**.264**
**Material (main)**	**35.477 (1, 13)**	<.001^***^	**.732**
Illumination × Material	1.958 (3, 39)	.136	.131
Illumination × Position	.241 (1.514, 19.683)	.726	.018
Material × Position	.342 (1, 13)	.569	.026
Illumination × Material × Position	.302 (3, 39)	.824	.023

^*^Indicates significance at the p < 0.05 level. ^***^Indicates significance at the p < 0.001 level.

**Table 7.** Results of ANOVA on variable error (area of ellipse fit to two-dimensional Gaussian).

Effect	*F* (*df*)	*p*	$\eta_{p}^{2}$
Illumination (main)	1.877 (2.356, 30.631)	.165	.126
**Position (main)**	**19.662 (1, 13)**	.001^**^	**.602**
Material (main)	.017 (1, 13)	.899	.001
Illumination × Material	.866 (4, 52)	.491	.062
Illumination × Position	1.565 (4, 52)	.197	.107
Material × Position	.539 (1, 13)	.476	.040
Illumination × Material × Position	.683 (1.963, 25.520)	.512	.050

^**^Indicates significance at the p < 0.01 level.

In support of our primary hypothesis, a main effect of material on CCIs was once again found, with a higher CCI for scenes containing glossy (mean = .391, *SD* = .282) than matte shapes (mean = .331, *SD* = .295). There were no significant interactions between material and position or illumination on the CCIs, against our second hypothesis. In addition, this time there was no significant main or interaction effects of material on the variable error.

There were no significant main or interaction effects of illumination on either CCIs or variable error, against our final hypothesis that scenes illuminated by daylight illuminants would result in higher CCIs.

A significant main effect of position was found on both CCIs and variable error. There was a higher CCI when the patch was on the wall (mean = .385, *SD* = .263) than on the shape (mean = .337, *SD* = .313), as in Experiment 2. To test whether the difference in local surround could explain this finding, we modeled the CCIs that an ideal observer would achieve using only local surround information on either the back wall or the shape. Unlike in Experiment 2, the shape containing the matching patch now changes reflectance on every trial. Therefore, the mean predicted settings over all six reflectances were averaged before calculating a predicted CCI on the shape. As before, CCIs on the wall were predicted to be higher (0.980) than on the shape (0.967). However, these are a lot more similar than in Experiment 2, which may explain why the effect size is smaller ($\eta_{p}^{2}=.264$ in Experiment 3 vs. $\eta_{p}^{2}=.296$ in Experiment 2).

In addition, there was more variable error when the patch was on the shape (mean = $1.979\times 10^{-4}$, *SD* = $1.455\times 10^{-4}$) than on the wall (mean = $1.238\times 10^{-4}$, *SD* = $8.87\times 10^{-5}$). To further explore this, we analyzed the variable error separately in u′ and v′. A significant main effect of position was found both on u′ $\left(F\left(1,13\right)=20.850,p=0.001,\eta_{p}^{2}=.616\right)$ and v′ $\left(F\left(1,13\right)=23.754,p<0.001,\eta_{p}^{2}=.646\right)$. Variable error in u′ was higher when the patch was on the shape (mean = $7.08\times 10^{-3}$, *SD* = $3.07\times 10^{-3}$) than on the wall (mean = $5.21\times 10^{-3}$, *SD* = $2.18\times 10^{-3}$). This pattern was also replicated in v′ with a higher variable error on the shape (mean = $9.83\times 10^{-3}$, *SD* = $3.52\times 10^{-3}$) than on the wall (mean = $8.02\times 10^{-3}$, *SD* = $2.99\times 10^{-3}$).

### 4.3 Interim discussion

The findings of this experiment are generally in agreement with those of Experiment 2. Again, we found evidence that observers are able to benefit from the presence of specular highlights, as shown by an increase in CCI. In fact, the effect was even stronger here, suggesting the effect of specular highlights found in Experiment 2 may have been underestimated. In the present experiment, observers were required to make more fine-tuned settings, resulting in more precise estimates, giving us more power to detect the true effect of highlights. However, there was still no decrease in the variability of settings for scenes containing glossy, compared to matte, shapes. In addition, the interaction between material and position on variable error, which was found in Experiment 2 was no longer significant (*p* = 0.071). The lack of an interaction between material and position or illumination suggests that the use of specular highlights is not mediated by either of these factors.

The CCIs were generally higher than in Experiment 2, which could be due to observers using the smallest step size for each achromatic setting. This encouraged observers to make settings closer to their true perceived gray, compared to in the previous experiments. Furthermore, this resulted in less variability when observers were making settings on the back wall. It should be noted that the variability when the matching patch was on the central shape remained high as the reflectance of the shape, and thus the local surround, changed on every trial.

As in both Experiments 1 and 2, there appears to be no benefit for scenes illuminated by daylights as opposed to nondaylights, against our final hypothesis, and previous research (e.g., Delahunt & Brainard, 2004). As there were no effects of illumination found on CCIs in any of the experiments, we ran one more analysis to determine whether this was due to lack of power. For this, the data from Experiments 2 and 3 were combined for a total of 28 observers. It should be noted that the data from Experiment 1 were not included in this analysis as the methods used were so different from the other experiments that it would not be appropriate to combine the data. A 4 (illumination) × 2 (position) × 2 (material) × 2 (experiment) ANOVA on the combined data still found no significant main effect of illumination (*p* = 0.688), but the effects of material (*p* < 0.001) and position (*p* = 0.004) remained significant. This suggests the lack of effect of illumination found in any individual experiment was not due to a lack of power.

The similarity of findings in this experiment and Experiment 2 suggests that the findings are not specific to one particular scene with certain reflectances. In addition, the fact that raw settings are still drawn toward the blue illumination chromaticity suggests that this is not caused by the local surround of the central shape, which previously always had the same surface reflectance but here averages out to a neutral chromaticity.

## Discussion

**Figure 13.**
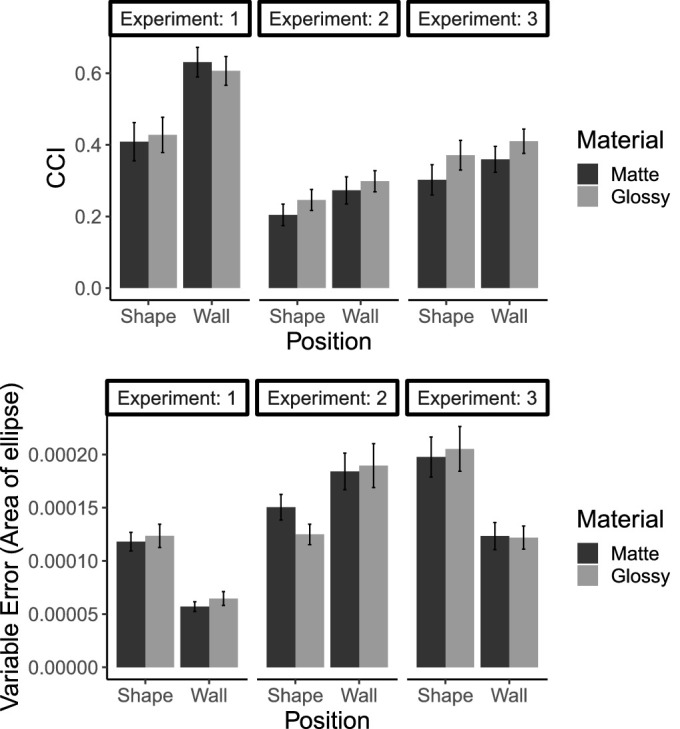
Summary results across all three experiments. Top: mean CCIs for matching patch on shape and wall, with matte or glossy shapes. Bottom: mean variable error, as measured by the area of an ellipse fit to a Gaussian. For both graphs, error bars show ± 1 standard error of the mean.

This series of experiments was designed to test three primary hypotheses: that specular highlights would improve color constancy and decrease variable error, that the effect of highlights would be mediated by the type of illumination (daylight or nondaylight) and position of a matching patch, and that scenes illuminated by daylights would result in a higher degree of color constancy than scenes illuminated by nondaylights. A summary of findings across all experiments, in terms of both variable error and CCIs, is shown in Figure 13. Observers had higher CCIs when making achromatic settings on the back wall than on the shape in all three experiments. In addition, in Experiments 2 and 3 (when a Mondrian background was introduced), CCIs were higher for scenes containing glossy, compared to matte, shapes. In both Experiments 1 and 3, estimates were less variable when the matching patch was on the back wall compared to on the shape. In Experiment 2, this effect was reversed, with higher variable error on the wall than on the shape. In addition, there was an interaction between material and patch position on variable error in Experiment 2.

In support of our primary hypothesis, we found highlights to improve color constancy in Experiment 2, when scenes contained a Mondrian background, in agreement with previous research (Yang & Shevell, 2002; Lee & Smithson, 2016). This finding was replicated with another group of observers in Experiment 3, when the reflectance of the shapes was changed on each trial. However, there was no effect of specular highlights when there was a uniform background (Experiment 1). The uniform light gray background in Experiment 1 could have provided a strong cue to the illumination, in terms of both local and global adaptation, thereby weakening reliance on specular highlights. Removing this cue resulted in an overall reduction in CCIs, allowing an otherwise masked effect of specular highlights to be revealed, in line with Xiao et al. (2012). While observers could theoretically have used the global mean or local surround cue with the Mondrian background, it seems that they found it more difficult to use such cues for color constancy. This is in contrast with previous research (Linnell & Foster, 2002) suggesting that having multiple surfaces of different reflectances in the scene (as in our Mondrian backgrounds) should improve color constancy. However, it should be noted that observers were probably using the global and local mean and brightest-is-white cues to some extent in Experiments 2 and 3, as shown by the nonzero CCIs for scenes containing matte shapes.

While it could be argued that the benefit seen for scenes containing specular highlights could be explained simply by the difference in local surround, there are at least three reasons to believe this not to be the case. When modeling the effect of local surround (subtending roughly 0.5 degrees from the matching patch), we found no difference between matte and glossy shapes. If the region of local surround were increased to include the specular highlights when the patch was on the shape, it could still not explain the increase in CCIs we see for scenes containing glossy shapes when the matching patch is on the back wall. Furthermore, if observers were simply using the local surround, we would expect to find the same results when the matching patch is on the shape in Experiments 1 and 2. The fact that there is no benefit for specular highlights with a uniform background suggests that observers are using more information than simply the local surround.

There was no overall reduction in variable error for scenes containing specular highlights on any of the three experiments. While we did find a Position × Material interaction in Experiment 2, this was not robust enough to show up in Experiment 3. This is against our prediction, based on the cue combination literature (Ernst & Banks, 2002; Ernst, 2006), that adding valid cues should decrease variability of estimates. A possible explanation for this null finding is that the variable error is sensitive to the number of trials, and nine per condition may be insufficient. Due to the many conditions in these experiments, it would be unfeasible to add more repeats per condition as each experiment already lasts an hour. Future experiments using more trials per condition, but fewer conditions, are needed to determine whether an effect of specular highlights on variability of estimates can be found.

In support of our second hypothesis, there was an interaction between material and position on variable error in Experiment 2, such that specular highlights only reduced variance when estimates were made on the shape and not the back wall. While this lends support to the notion that scenes are separated into different illumination frameworks and agrees with Yang and Maloney (2001), the finding was not replicated in Experiment 1 or 3, so should be taken with caution. Furthermore, there were no such interactions on CCIs in any of the three experiments. Therefore, our results do not strongly support the prediction that the effect of specular highlights on constancy will be mediated by patch position.

In addition, there were no significant interactions between specular highlights and illumination, suggesting the effect is also not mediated by type of illumination. This is in contrast to Yang and Maloney (2001), who found observers only used specular highlights when they signaled a daylight illumination. However, it should be noted that the illuminations used in the present study were more controlled, such that they were all equally discriminable from neutral. Furthermore, as highlights were not perturbed in the present study, all scenes were physically possible—even those illuminated by nondaylights—which may explain the lack of interaction found here.

Against our final hypothesis, there were no significant effects of illumination, even when data from Experiments 2 and 3 were pooled. Unlike Delahunt and Brainard (2004), we did not find an improvement in color constancy for scenes illuminated by daylights compared to nondaylight illuminants. However, it should be noted that in Delahunt and Brainard (2004), the effect of illumination was only statistically significant when a local surround cue was silenced. While the specular highlight cue was absent in half the trials of the present experiments, there were no inconsistent cues, and the global mean and brightest-is-white cues were always present and consistent.

While there were no effects of illumination on the equivalent illuminant calculations, a robust finding was a bias in raw achromatic settings toward blues. This was not an artifact of the local surround, as in Experiment 3, the local surround on the shape was varied on a trial-by-trial basis. A plausible explanation for this bias is that observers assume illuminations are bluish. Therefore, when they see a surface containing more shorter wavelengths, they attribute the extra blue to the illumination and discount it in their achromatic settings.

A further finding was the overall effect of patch position on CCIs. In all three experiments, observers had a higher degree of color constancy when the matching patch was on the back wall than on the shape. This was most likely due to the difference in local surround, rather than differences in local lightness contrast between the patch and surround across positions. In Experiments 2 and 3, the patch luminance was lower than that of the surround in both positions, and the absolute luminance contrast was greater on the wall than the shape, which should have resulted in lower CCIs on the wall, counter to what we found. In fact, the effect we found was qualitatively predicted by modeling the effect of local surround.

Over all three experiments, but particularly in Experiments 2 and 3, we found CCIs lower than reported in many previous studies. There are a number of possible explanations for the low CCIs found here. In many of the previous studies, the environment was more immersive than here—either using an immersive room (Gupta, Gross, Pastilha, & Hurlbert, 2020) or stereoscopic viewing (Yang, Kanazawa, Yamaguchi, & Kuriki, 2013; Delahunt & Brainard, 2004; Xiao et al., 2012). Yang and Shevell (2002) found that binocular disparity, achieved through stereoscopic viewing, improves color constancy, with CCIs under monocular viewing similar to those found here. Furthermore, there are large individual differences in CCIs, with nonnaive observers often outperforming naive observers. Here we used only naive observers, which may have contributed to the lower CCIs. Finally, the specific setup of the stimuli used, as well as the instructions (Arend & Reeves, 1986), can have a large impact on the resulting degree of color constancy. Indeed, this is what we found here, with much lower CCIs when a Mondrian background wall was introduced, as opposed to a uniform, spectrally neutral wall.

Many of the previous studies into the use of specular highlights as a cue are not suitable for formal statistical analyses and did not consider how specular highlights may interact with other factors. Here, we increased the number of observers and extended previous findings that observers are able to use specular highlights. However, this was only the case when a uniform background cue was weakened. Furthermore, controlling the illuminations, such that those on and off the daylight locus were equally discriminable, removed the interaction between illumination and specular highlights found previously (Yang & Maloney, 2001). We did not find that observers used a daylight prior to improve color constancy estimates. The novel cue combination approach taken here revealed some interesting results that would have otherwise been overlooked. However, further studies investigating the effect of cues on variable error in color constancy using different methods, such as asymmetric matching, or using more immersive environments, are needed to draw firmer conclusions. Additionally, using the cue combination approach to study how variable error is affected by other cues (such as global mean), which we did not manipulate here, would be of great interest.

## Supplementary Material

Supplement 1

## Appendix

### Instructions

“In the following trials, you will see an image of a scene. In each scene, there are six randomly colored shapes sitting in a box. On top of the central shape (on one wall) you will see a small patch, indicated by a black outline. Your task is to adjust the color of this patch so that it looks like a grey piece of paper sitting on top of the shape in the scene. That is, adjust the patch so that it looks like it has the same reflectance properties as a grey surface would in this scene. You may notice an overall color change of the scenes on some trials. Think of this as a change to a different color of illumination and focus on adjusting the patch so that it looks like it has the same surface reflectance as a grey surface. That is, adjust the patch so that it looks like a grey surface under the changed illumination.”

**Table A.1.** The sets of Munsell chips used for the six blobby shapes in Experiments 1 and 2. In Experiment 3, only Set 1 was used.

Set 1	Set 2	Set 3
5BG4/6	5BG4/6	5BG4/6
10B3/4	2.5Y7/6	10Y7/8
10Y5/6	7.5B3/6	10RP5/8
2.5PB4/8	10YR6/8	5PB5/10
5YR5/8	2.5RP5/10	10B5/8
2.5P4/4	7.5PB5/6	10YR5/4
