# Supplementary material for: Specular highlights improve color constancy when other cues are weakened
Source: J Vis. 2020 Nov 10;20(12):4. doi: 10.1167/jov.20.12.4 (PMC7674000; doi:10.1167/jov.20.12.4)
Supplement: Supplement 1 [file jovi-20-12-4_s001.pdf]

Supplementary Material

To ensure there were no differences in cue validity between the matte and glossy scenes used, we modelled the chromaticity which would be predicted using the global mean and brightest is white cues. We did not model the local surround for these purposes as this was not affected by the specularity of the shapes, and was modelled separately. We modelled the chromaticity predicted by these cues for one scene illuminated by each of the five illuminants used Experiments 1 and 2. It was not necessary to perform this modelling for Experiment 3 as the stimuli were so similar to those used in Experiment 2.

To model global mean chromaticity, we took the average chromaticity of all pixels in the scene. For the glossy shapes this included the specular highlights. This is shown by the circles in Fig S1. To model brightest is white, we identified the pixel with the highest luminance (Y), and calculated the chromaticity of this pixel. For both Experiments 1 and 2, with matte and glossy scenes this was a pixel on the back wall. This is shown by the diamonds in Fig S1. In addition, we modelled the chromaticity convergence cue for the glossy scenes. To do this, we identified 40 pixels lying on a line on each shape which included a specular highlight and the body colour. For each of the six shapes, a line was fit to the chromaticities of these 40 pixels, and the intersection of the six lines was calculated. This chromaticity is shown by the Xs in Fig S1

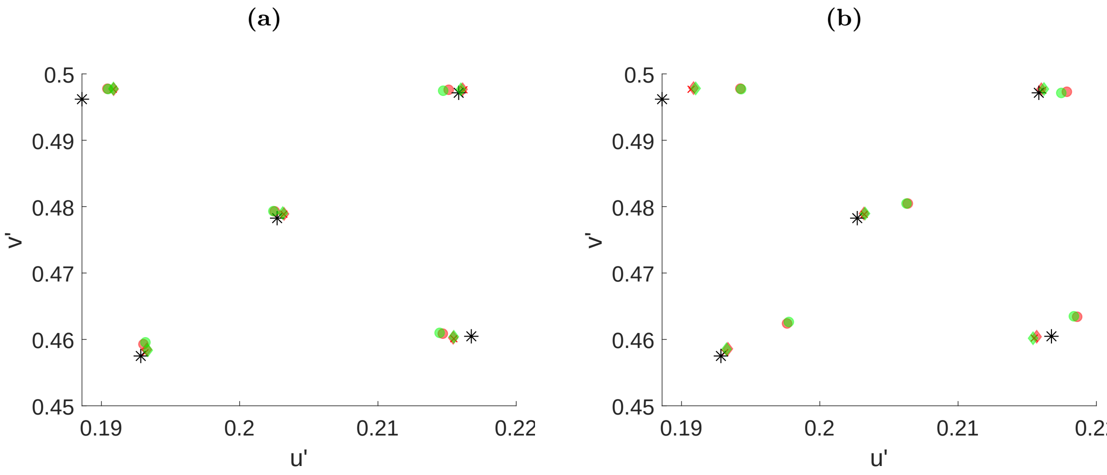

Figure S1: chromaticity implied by various cues. Black *s are illumination chromaticity; green symbols are cues in matte scenes, red symbols are cues in glossy scenes. Circles are global mean; diamonds are the brightest point; red Xs are chromaticity convergence (in glossy scenes only). (a) is Experiment 1; (b) is Experiment 2.

To ensure truncating the out of gamut pixels did not affect the chromaticity of the specular highlights, we plotted the rendered scenes with the truncated pixels shown in black. This can be seen in Fig S2, for Experiment 1 on the top row and Experiment 2 on the bottom row. None of the truncated pixels coincide with the specular highlights.

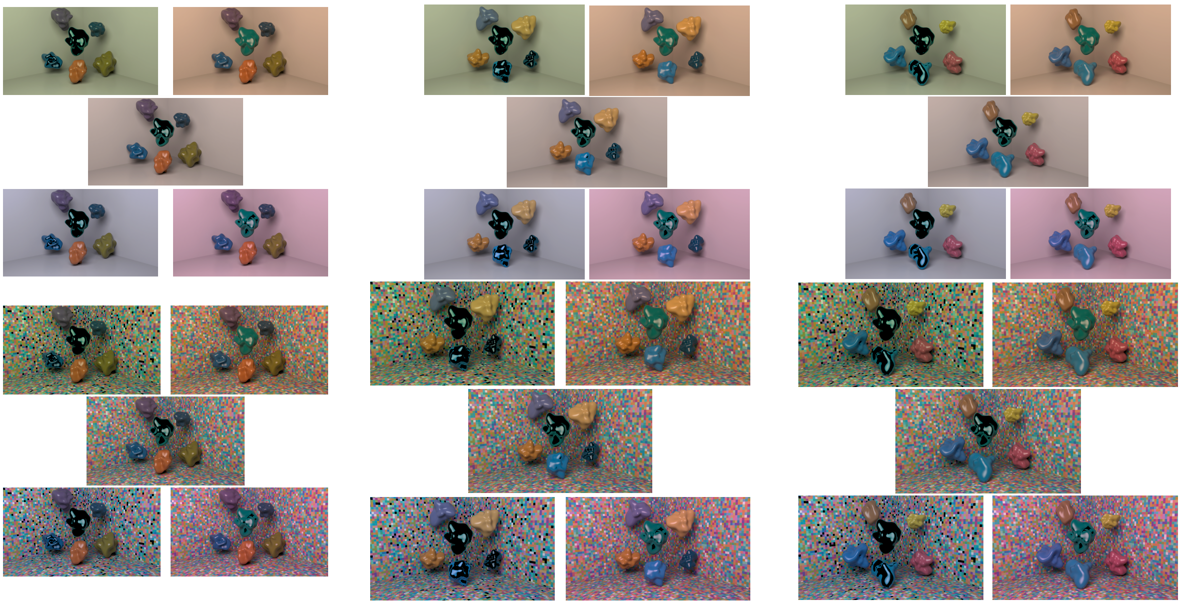

Figure S2: The scenes used in Experiment 1 (top) and 2 (bottom), with the location of the truncated pixels shown in black. The scenes used in Experiment 3 are not shown, as the reflectances are the same as those used in the leftmost image of experiment 2.
